# Low NaCl Concentrations Increase Cotyledon Growth in Chinese White Radish (*Raphanus sativus* L. var. *longipinnatus* Bailey) Seedlings via Aquaporin-Mediated Water Transport

**DOI:** 10.3390/plants14111616

**Published:** 2025-05-26

**Authors:** Nutkamol Masepan, Sitthisak Intarasit, Aussara Panya, Jarunee Jungklang

**Affiliations:** 1 Department of Biology, Faculty of Science, Chiang Mai University, Chiang Mai 50200, Thailand; nutkamol_m@cmu.ac.th (N.M.); sitthisak.inta@cmu.ac.th (S.I.);; 2 Ph.D.’s Degree Program in Biology (International Program), Faculty of Science, Chiang Mai University, Chiang Mai 50200, Thailand

**Keywords:** *Raphanus sativus*, low NaCl concentration, growth, water status, aquaporin genes, ion contents

## Abstract

This study aimed to elucidate the specific role of low NaCl concentrations, particularly 10 and 20 mM, in stimulating cotyledon growth in Chinese white radish (*Raphanus sativus* L. var. *longipinnatus* Bailey) seedlings. Chinese white radish seeds were cultivated in sand culture and subjected to daily watering with solutions containing 0, 10, 20, 50, or 100 mM NaCl. Growth, water status, aquaporin gene expression, ion contents, and physiology-related parameters were assessed 4 days after sowing. Applying 10 and 20 mM NaCl significantly promoted the growth of 4-day-old seedlings. Notably, the cotyledons exhibited the most significant growth, achieving a rate of 177% compared with the 125–138% growth observed in the hypocotyl and root parts. This substantial enhancement in cotyledon growth, including biomass, cotyledon area, cotyledon thickness, and mesophyll cell size, was induced by an optimal concentration of 10 mM NaCl. This induction correlated with the increased water content, degree of succulence, and expression of aquaporin genes, specifically within *PIP1-1*, *PIP1-2*, *PIP2-1*, *PIP2-2*, and *TIP1-1*, in addition to the maintenance of the Hill reaction, heightened free radical scavenging, and the elevated accumulation of Na^+^, Cl^−^, K^+^, proline, total N, and C. These findings suggest a beneficial role of low NaCl levels in optimising early-stage seedling growth.

## 1. Introduction

In general, elevated NaCl concentrations in soil can lead to ionic imbalance, osmotic stress, and oxidative stress, resulting in reduced plant growth and development [1]. Conversely, lower NaCl concentrations (<50 mM) have been shown to have positive effects on the growth and development of certain plant species, especially halophytes. However, research on the effects of low NaCl concentrations on plants has been less extensive compared to high NaCl levels. The application of low NaCl concentrations can produce suitable levels of sodium ions (Na^+^) and chloride ions (Cl^−^), which can act as beneficial elements and osmotica, thereby enhancing plant growth through various developmental processes observed in numerous plant species. For example, *Arabidopsis thaliana* exhibited increased shoot and root biomass when exposed to a low concentration of 5 mM NaCl, accompanied by elevated sulfur (S), carbon (C), zinc (Zn), and copper (Cu) contents [2]. Supplying Na^+^ to highly salt-tolerant sugar beets increased their leaf area, stomatal density, and shoot fresh weight [3]. Cl^−^ has been shown to stimulate fresh weight growth by enhancing cell expansion in leaves and cell elongation in tobacco leaves and roots, which is attributed to its role as an osmoregulator that enhances water use efficiency and maintains turgor pressure within the cell [4]. Similarly, Cl^−^ accumulation in the shoots of *Pugionium cornutum* induces turgor pressure, thereby enhancing the plant’s osmotic adjustment ability under saline and drought conditions [5]. These plants employ various strategies to enhance water use efficiency, including the utilisation of osmoregulators, maintenance of ion homeostasis, and regulation of water flux [6].

Aquaporin (AQP) proteins play a crucial role in regulating water influx and efflux in plant cells. AQPs are membrane channel proteins that play a key role in transporting water, hydrogen peroxide, and other small molecules, such as CO_2_, NO, glycerol, ammonia, urea, and boron. They are categorised into five major subfamilies within higher plants: plasma membrane intrinsic proteins (PIPs), tonoplast intrinsic proteins (TIPs), nodulin 26-like intrinsic proteins (NIPs), small basic intrinsic proteins (SIPs), and uncategorised intrinsic proteins (XIPs). In plants, PIPs and TIPs facilitate water translocation across cellular membranes and subcellular compartments [7,8,9]. AQPs are involved in drought and salt stress tolerance in several plant species. For instance, *MaPIP1-1*, known as an AQP gene in banana, exhibited overexpression in transgenic *Arabidopsis* plants under 50–150 mM NaCl conditions, leading to enhanced primary root length, increased root hair development, reduced ion leakage and malondialdehyde (MDA) content, decreased cytosolic K^+^ and Na^+^ accumulation, and an improved K^+^/Na^+^ ratio compared to wild-type seedlings [10]. The upregulation of *SsPIP* expression in the halophytic plant *Suaeda salsa* L. under 100 mM NaCl treatment correlated with increased shoot fresh weight, osmotic potential, root hydraulic conductance, and succulence degree [11]. However, the impact of low NaCl concentrations on AQP expression in plant cells has received less attention compared to high NaCl concentrations.

The seedling or sprout stage of Chinese white radish (*Raphanus sativus* L. var. *longipinnatus* Bailey) is rich in vitamins (B1, B2, B3, B5, B6, B9, and C) and minerals (Cl, K, Zn, Fe, Mg, Mn, and P), and contains bioactive compounds such as polyphenols and glucosinolates. These seedlings have been recommended for consumption due to their high nutritional value and the presence of anti-cancer compounds that contribute to human health [12,13]. To date, this sprout has been distributed on the shelves of numerous supermarkets across Thailand. Chinese white radish has been identified as a halophyte exhibiting moderate tolerance to salinity stress, as it is capable of surviving and completing its life cycle under high-salinity conditions (0.61–1.2 dS/m EC 1:5), which corresponds to approximately 0.4–0.8% soil salinity [14,15]. Seven-day-old seedlings of this species can also grow under saline conditions (0.4–0.8 dS/m EC 1:5 in sand culture) [16]. Yuan et al. [17] reported that treatment with 10 mM NaCl increased the yields of 5- and 7-day-old radish seedlings by 50% and 64%, respectively, while treatment with 50 mM NaCl resulted in yield increases of 74% and 100%, respectively.

Despite these findings, the effects of low NaCl concentrations on the growth of Chinese white radish seedlings remain relatively unexplored. This study aims to elucidate the positive effects of a low NaCl concentration on increasing growth, particularly in the cotyledon, in 4-day-old Chinese white radish seedlings. We hypothesised that low NaCl concentrations would promote seedling growth by enhancing water influx into cotyledon cells, thereby increasing water status, maintaining ion homeostasis, and ultimately promoting cotyledon growth. This research contributes to the understanding of how reduced NaCl levels stimulate the growth of Chinese white radish seedlings and may offer insights into enhancing seedling viability, particularly under environmental stresses such as salinity and water deficit.

## 2. Results

### 2.1. Effects of Low to High NaCl Concentrations on Growth of Chinese White Radish Seedlings

**Figure 1:**
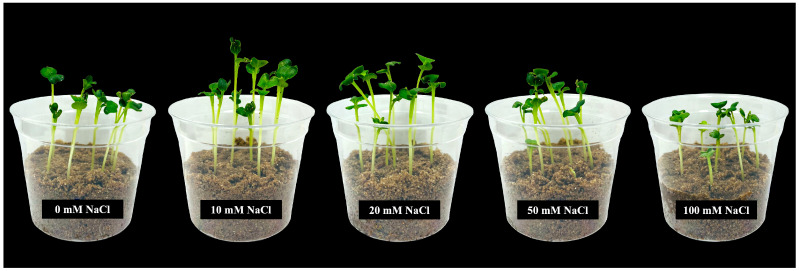
Effects of 0, 10, 20, 50, and 100 mM NaCl on growth of 4-day-old Chinese white radish (*Raphanus sativus* L. var. *longipinnatus* Bailey) seedlings.

**Figure 2:**
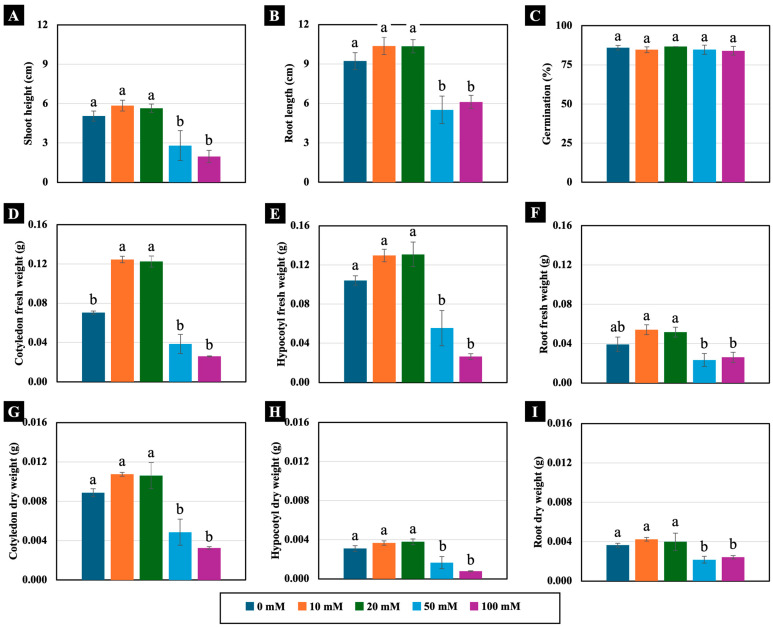
The effects of 0, 10, 20, 50, and 100 mM NaCl on the shoot height (**A**), root length (**B**), germination (**C**), fresh weight (**D**–**F**), and dry weight (**G**–**I**) of 4-day-old Chinese white radish seedlings. The results are the means of the three replications ± standard deviation (SD). Different letters (a and b) for the same parameter indicate significant differences at *p* < 0.05, determined by Duncan’s multiple range test.

All the tested NaCl concentrations (10, 20, 50, and 100 mM) did not significantly inhibit seed germination or seedling growth in 4-day-old Chinese white radish. However, seedling growth rates were observed to be slower and unevenly germinated under moderate (50 mM) and high (100 mM) NaCl concentrations compared to the control (Supplementary Figure S1). Specifically, the 50 and 100 mM NaCl treatments led to reductions in shoot height, root length, and hypocotyl fresh weight, as well as cotyledon, hypocotyl, and root dry weight compared to those of the control (Figure 1 and Figure 2). In contrast, lower NaCl concentrations (10 and 20 mM) did not negatively affect these growth parameters. Instead, they resulted in a significant increase in cotyledon fresh weight, with enhancements of up to 177% and 178%, respectively, compared to those of the control (Figure 2D).

### 2.2. Effects of Low NaCl Concentrations on Structure and Function of Chinese White Radish Cotyledons

#### 2.2.1. Growth Parameters

**Figure 3:**
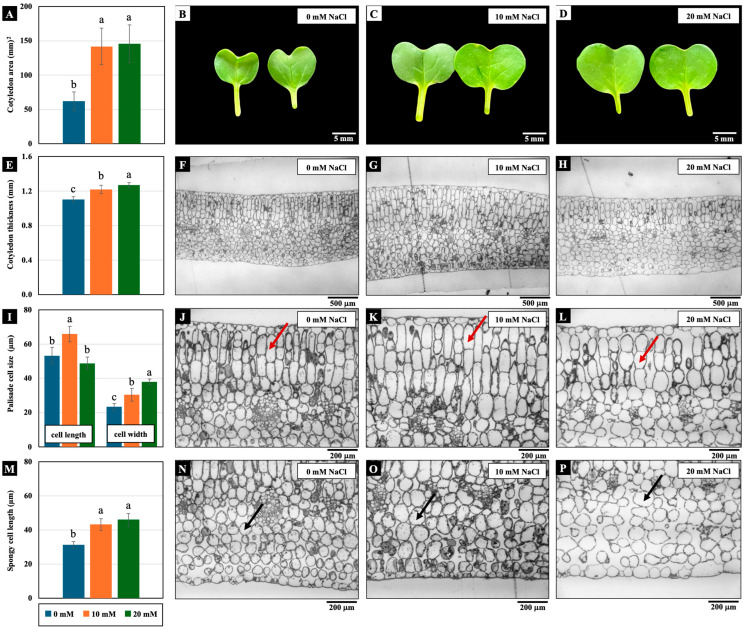
The effects of 0, 10, and 20 mM NaCl on the cotyledon area (**A**–**D**), cotyledon thickness (**E**–**H**), palisade (red arrow) mesophyll cell size (**I**–**L**), and spongy (black arrow) mesophyll cell width (**M**–**P**), respectively, of the cotyledons of 4-day-old Chinese white radish seedlings. The results are the means of three replications ± standard deviation (SD). Different letters (a–c) for the same parameter indicate significant differences at *p* < 0.05, determined by Duncan’s multiple range tests.

Low NaCl concentrations (10 and 20 mM) affected cotyledon growth. The cotyledon area, cotyledon thickness, palisade cell width, and spongy cell length were significantly increased by these NaCl concentrations compared to the control. In addition, only the palisade mesophyll cell length of the cotyledons treated with 10 mM NaCl increased significantly (Figure 3).

#### 2.2.2. Water Status and Aquaporin Gene Expression

**Figure 4:**
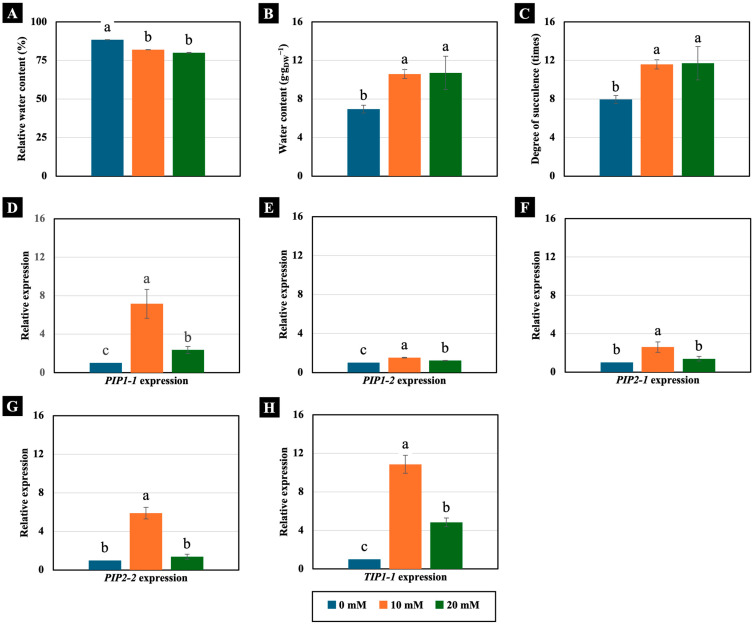
The effects of 0, 10, and 20 mM NaCl on the relative water content (**A**), water content (**B**), degree of succulence (**C**), and expression of the *PIP1-1* (**D**), *PIP1-2* (**E**), *PIP2-1* (**F**), *PIP2-2* (**G**), and *TIP1-1* (**H**) genes, respectively, in the cotyledons of 4-day-old Chinese white radish seedlings. The results are the means of three replications ± standard deviation (SD). Different letters (a–c) for the same parameter indicate significant differences at *p* < 0.05, determined by Duncan’s multiple range test.

Concentrations of 10 and 20 mM NaCl had different effects on the water status of 4-day-old Chinese white radish seedlings. Although they significantly decreased the RWC of the cotyledons (Figure 4A), they conversely increased the water content and degree of succulence. Notably, this increase exceeded 150% compared to the control (Figure 4B,C).

Moreover, these low NaCl concentrations increased the expression of AQP genes, which are ubiquitous membrane channel proteins that play key roles in water transport across membranes. Treatment with 10 mM NaCl significantly increased the expression of the *PIP1-1*, *PIP1-2*, *PIP2-1*, *PIP2-2*, and *TIP1-1* genes, while the 20 mM NaCl treatment significantly increased the expression of the *PIP1-1*, *PIP1-2*, and *TIP1-1* genes. However, the most remarkable increase in expression was observed in the *TIP1-1* gene, which rose by 1084% and 483%, respectively, compared to the control (Figure 4D–H).

#### 2.2.3. Ion Contents

**Figure 5:**
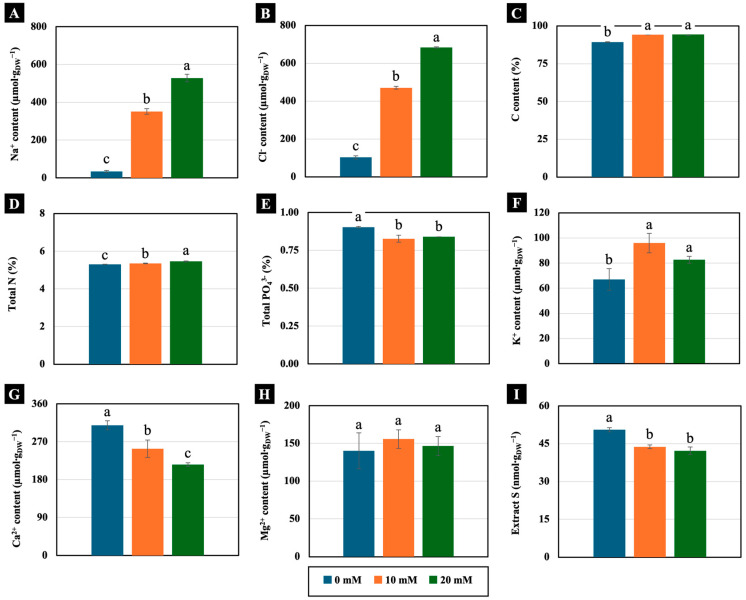
The effects of 0, 10, and 20 mM NaCl on the Na^+^ content (**A**), Cl^−^ content (**B**), C content (**C**), total N (**D**), total PO_4_^3−^ (**E**), K^+^ content (**F**), Ca^2+^ content (**G**), Mg^2+^ content (**H**), and extract S (**I**), respectively, of the cotyledons of 4-day-old Chinese white radish seedlings. The results are the means of three replications ± standard deviation (SD). Different letters (a–c) for the same parameter indicate significant difference at *p* < 0.05, determined by Duncan’s multiple range test.

Low NaCl concentrations (10 and 20 mM) significantly increased the accumulation of Na^+^ and Cl^−^ in the cotyledons of 4-day-old Chinese white radish seedlings compared the control. In the cotyledons of the seedlings treated with 10 and 20 mM NaCl, Na^+^ accumulation was more than 1000% higher, whereas Cl^−^ accumulation was more than 450% greater compared to the control (Figure 5A,B). Additionally, these low concentrations exhibited distinct trends in the macronutrient content of the cotyledons of 4-day-old Chinese white radish seedlings. The levels of C, total N, and K^+^ increased significantly in cotyledons treated with 10 and 20 mM NaCl. The K^+^ content increased by over 120% compared to the control (Figure 5C,D,F). Conversely, the contents of PO_4_^3−^, Ca^2+^, and extract S decreased in the cotyledons treated with low NaCl. Specifically, both the Ca^2+^ and extract S contents were reduced by more than 18% compared to the control (Figure 5E,G,I). However, the Mg^2+^ content was not significantly different between the treated and untreated seedlings (Figure 5H).

#### 2.2.4. Physiology-Related Parameters

**Figure 6:**
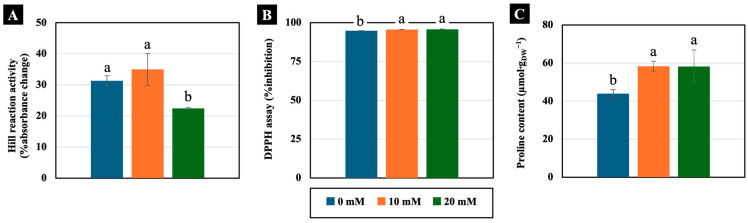
The effects of 0, 10, and 20 mM NaCl on Hill reaction activity (**A**), free radical scavenging activity by DPPH assay (**B**), and proline content (**C**), respectively, in the cotyledons of 4-day-old Chinese white radish seedlings. The results are the means of three replications ± standard deviation (SD). Different letters (a and b) for the same parameter indicate significant differences at *p* < 0.05, determined by Duncan’s multiple range test.

Low NaCl concentrations (10 and 20 mM) had varying effects on physiology-related parameters. The Hill reaction, which represents the activity of the electron transport system, was not significantly different in the cotyledons treated with 10 mM NaCl for 4 days. However, this function was reduced in the cotyledons treated with 20 mM NaCl (Figure 6A). Additionally, 10 and 20 mM NaCl slightly increased the free radical scavenging activity, assessed using the DPPH assay, measuring 101% compared to the control in the cotyledons of 4-day-old Chinese white radish seedlings (Figure 6B). In contrast, proline content significantly increased with increasing NaCl concentration, showing levels of up to 133% of that of the control in the cotyledons (Figure 6C).

#### 2.2.5. Correlation Coefficients Among Cotyledon Parameters Under Low NaCl Concentrations

**Figure 7:**
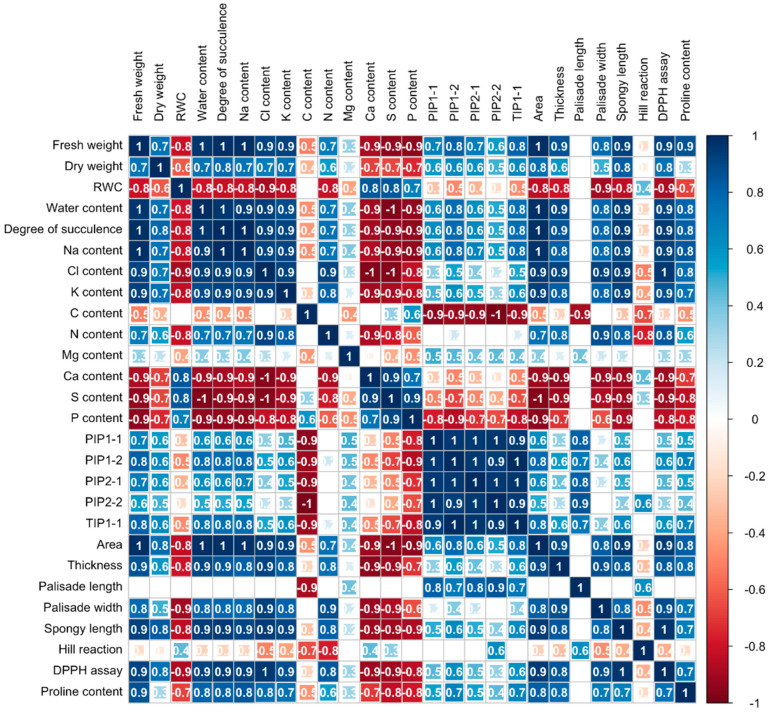
Correlation coefficients among cotyledon parameters under low NaCl concentrations in 4-day-old Chinese white radish seedlings. Pearson’s correlation coefficient (r) significant at *p* < 0.05.

The correlation coefficients among various parameters in the cotyledons of 4-day-old Chinese white radish exhibited considerable variability. Fresh weight demonstrated a positive correlation with dry weight, water content, degree of succulence, sodium, chloride, potassium, nitrogen, the expression of aquaporin genes (*PIP1-1*, *PIP1-2*, *PIP2-1*, and *TIP1-1*), area, thickness, palisade cell width, spongy cell length, free radical scavenging activity measured by DPPH assay, and proline content in the cotyledons. Conversely, fresh weight showed a negative correlation with relative water content (RWC), carbon, calcium, phosphorus, and sulfur levels. Furthermore, sodium exhibited a positive correlation with potassium and the expression of aquaporin genes, while both sodium and chloride displayed negative correlations with calcium, phosphorus, and sulfur levels (Figure 7).

## 3. Discussion

This study employed moderate (50 mM) and high (100 mM) concentrations of NaCl, which reduced growth in 4-day-old Chinese white radish seedlings by decreasing shoot height, root length, fresh weight, and dry weight, but low concentrations of 10 and 20 mM NaCl resulted in significant enhancements in growth, as measured by cotyledon fresh weight (Figure 1 and Figure 2). These observed effects can be attributed to the slight accumulation of soluble salts in sand culture due to the low levels of NaCl concentration, thereby creating optimal conditions for seedling development. This assertion is supported by the preliminary experiment (Supplementary Figure S2), where the electrical conductivity (EC) in the sand culture watered with 10 and 20 mM NaCl solutions ranged from 0.08 to 0.15 dS/m over a period of 0–4 days. These values indicate a slight salinity in the sand culture [14,15,18], providing an optimal supply of Na^+^ and Cl^−^ ions in the sand culture, which serve as essential nutrients for these seedlings, while 50 and 100 mM NaCl, which had the higher EC values in watered sand culture, ranged from 0.21 to 0.35 (moderate salinity) and 0.40 to 0.65 dS/m (strong salinity), respectively. These concentrations may represent higher doses that are not optimal for promoting growth.

Previous studies have indicated that Na^+^ is a beneficial element for certain halophytic species, while Chinese white radish is a moderately salt-tolerant plant [19]. This ion shares chemical and structural similarities with K^+^ and can partially substitute K^+^ in various functions, facilitating the long-distance transport of anions and enhancing photosynthetic activity in these species [3,20,21]. Additionally, Cl^−^ is a plant micronutrient required in small quantities for various physiological processes. Cl^−^ plays a pivotal role in stabilising the photosynthetic reactions involved in oxygen evolution and cell division in both leaves and roots. Moreover, it is a regulator of specific enzyme activities, such as vacuolar proton-pumping ATPase activity [22,23]. Our findings align with Yuan et al. [17], who showed that radish seedlings aged 3–7 days and exposed to 10 mM NaCl exhibited increased fresh weight without reducing germination. Similarly, Hongqiao et al. [2] observed that 5 mM NaCl increased shoot and root biomass in 11-day-old *Arabidopsis thaliana* seedlings.

However, the increase in cotyledon fresh weight was remarkable among the three plant parts, with a significant increase of 177–178% compared to the control (Figure 2). This augmentation in fresh weight observed in the cotyledons of Chinese white radish under low NaCl concentrations can be ascribed to heightened cell expansion, signifying changes in mesophyll cell width and length. Consequently, this led to an expansion in the cotyledon area and thickness (Figure 3 and Figure 7). This phenomenon was closely associated with the enhancement of water status, as indicated by the plants’ water content, degree of succulence, and elevated levels of Na^+^, Cl^−^, and K^+^ ions and proline content (Figure 5, Figure 6C and Figure 7). In radish, spraying with proline treatment mitigated salinity stress by increasing the transpiration rate, stomatal frequency, leaf area, dry matter, water content, and photosynthetic pigments [24]. Proline is recognised as an osmotic solute pivotal in regulating the water balance between plant cells and the environment under osmotic and salinity stress [25,26,27]; Na^+^, Cl^−^, and K^+^ ions may serve as osmoregulators, mitigating the osmotic potential of seedling tissues. The role of Na^+^, Cl^−^, and K^+^ as osmotic agents facilitating osmotic adjustment in plants under salinity stress has been corroborated by prior investigations [5,21].

The exceptionally high accumulation of Na^+^ and Cl^−^ in the cotyledons of Chinese white radish may serve as a primary mechanism for facilitating water uptake into the cells (Figure 5A,B), consequently aiding in various metabolic processes, such as the hydrolysis of adenosine triphosphate (ATP), cell division, and photosynthesis, while also mitigating the toxicity of excess Na^+^ and Cl^−^ ions by diluting tissue concentrations [28,29]. Moreover, Cl^−^ benefits the growth of chloride-tolerant species, such as *Pugionium cornutum*, by enhancing leaf hydration, photosynthetic activity, and biomass [5]. Assessing RWC is crucial to gauge water insufficiency, as it reflects plant tissue hydration in a fully turgid state [26]. The RWC, which indicates the free energy of water within the cell (typically above 80%), signifies adequate hydration. Although our study revealed a 4–6% decrease in cotyledon RWC with low NaCl concentrations (Figure 4), it did not imply water stress, remaining within an acceptable range (above 80% RWC) for Chinese white radish seedlings and having no detrimental effect on their growth [30,31].

Aquaporin (AQP) genes play a crucial role in regulating water transport across cellular and organellar membranes, thereby influencing essential physiological processes in plants. These genes encode water channel proteins that facilitate the movement of water molecules, contributing to cellular hydration and turgor pressure, which are both vital for plant growth and development. Previous studies have identified *RsPIP1*, *RsPIP2*, and *RsTIP1* as the predominant AQPs expressed in radish seedling tissues. The *PIP1* and *PIP2* genes encode plasma membrane intrinsic proteins (PIPs) that mediate water transport across the plasma membrane, whereas *TIP* genes encode tonoplast intrinsic proteins (TIPs), which function at the vacuolar membrane [7]. Among these, specific members of the RsPIP2 group are upregulated in response to osmotic stress, while the RsPIP1 and RsTIP1 groups act as constitutive aquaporins [32]. Fricke and Knipfer [33] emphasised that proteins of the PIP and TIP families do more than facilitate water entry into individual cells; they play a pivotal role in enabling transcellular water flow, particularly in rapidly expanding tissues. This process is essential for maintaining turgor pressure, which is a key driver of continuous cell expansion and development. Supporting this, the constitutive overexpression of the *PtoPIP1-1* gene in *Arabidopsis* enhanced plant water status and turgor pressure, promoting cell expansion in both roots and leaves, ultimately accelerating bolting and flowering [34]. Furthermore, under prolonged high-salinity stress (100 mM NaCl for 10 days), *PIP1* expression in melon roots remained unchanged, while *TIP1-1* was significantly upregulated, suggesting its prominent role in maintaining turgor pressure and facilitating Na^+^ sequestration into vacuoles [35].

Our results revealed a notable upregulation of the *PIP1-1*, *PIP1-2*, *PIP2-1*, *PIP2-2*, and *TIP1-1* genes in cotyledons treated with 10 mM NaCl, whereas the 20 mM NaCl treatment induced the expression only of *PIP1-1*, *PIP1-2*, and *TIP1-1* (Figure 4 and Figure 7). These findings suggest that 10 mM NaCl more effectively stimulates the expression of specific AQPs, particularly those associated with the plasma and vacuolar membranes. According to previous reports, PIP1 proteins exhibit high water channel activity when coexpressed with PIP2 proteins, forming PIP1–PIP2 heterotetramers. This complex not only facilitates the trafficking of PIP1 proteins to the plasma membrane but also enhances PIP2 protein activity by increasing membrane water permeability [36]. The enhanced expression of AQP genes under 10 mM NaCl conditions appears to be closely associated with increased water channel function, thereby promoting cell expansion. Microscopic analysis revealed greater cell expansion and reduced intercellular spaces in cotyledon cross-sections under 10 mM NaCl treatment compared to those exposed to 20 mM NaCl (Figure 3). This morphological change corresponds with the upregulation of *PIP1-1*, *PIP1-2*, *PIP2-1*, *PIP2-2*, and *TIP1-1* (Figure 4), indicating that the lower salt concentration more effectively enhances AQP-mediated water transport. This improved water permeability likely facilitated rapid water movement across both the plasma and vacuolar membranes, thereby supporting turgor-driven cell expansion. The resulting increase in cell volume would enhance the availability of water and solutes necessary for key physiological functions, including photosynthesis, ultimately contributing to improved cotyledon growth in 4-day-old Chinese white radish seedlings.

The high accumulation of sodium ion (Na^+^) and chloride ion (Cl^−^) disrupts ion homeostasis by either increasing or decreasing the uptake of essential macronutrients [6]. This disruption can significantly affect plant growth and development, as macronutrients play crucial roles in various physiological processes. Macronutrients such as nitrogen (N), phosphorus (P), potassium (K), calcium (Ca), magnesium (Mg), and sulfur (S) are vital for plant health. N is a key component of proteins, enzymes, and chlorophyll, directly influencing photosynthesis and energy transformation. P contributes to ATP production, nucleic acids (DNA and RNA), and cell membrane structure. K is essential for metabolite transport, water balance, and stomatal regulation. Ca maintains cell wall integrity and acts as a signalling molecule for growth and stress responses. Mg, as the central atom in chlorophyll, plays a major role in photosynthesis and enzyme activation. S is necessary for chlorophyll synthesis, protein formation, and amino acid production, particularly for methionine and cysteine [22,37]. In fact, excessive Na^+^ accumulation primarily disrupts the uptake of cations such as Ca^2+^, K^+^, and Mg^2+^, whereas high Cl^−^ levels interfere with the uptake of anions like nitrate (NO_3_^−^), sulfate (SO_4_^2−^), and phosphate (PO_4_^3−^) [2,38]. This imbalance alters nutrient availability and may impair plant growth. The disruption of ion homeostasis has been observed in Chinese white radish seedlings. Exposure to 10 and 20 mM NaCl led to an increase in K^+^ and N content, while Ca^2+^, S, and P levels decreased. Interestingly, Mg^2+^ content remained unchanged (Figure 5). This suggests that these low NaCl concentrations selectively influence macronutrient uptake. Additionally, 10 mM NaCl induced greater K^+^ accumulation compared to 20 mM NaCl, correlating positively with water status (Figure 7). This finding implies that a low NaCl concentration (10 mM) may be more optimal for promoting cotyledon growth, particularly through water accumulation regulated by K^+^. K^+^ is known to enhance Na^+^ sequestration within leaf vacuoles, a key mechanism for maintaining cytosolic K^+^ retention, which is crucial for salt tolerance. Studies have shown that plants with higher cytosolic K^+^ concentrations exhibit improved resilience to NaCl stress [39,40]. Similarly, Cl^−^ accumulation disrupts NO_3_^−^, SO_4_^2−^, and PO_4_^3−^ uptake, leading to reductions in N, S, and P levels [2]. Despite the observed declines in S and P content in this study, N content increased under low NaCl concentrations (Figure 5). Previous research has reported similar findings, where NaCl treatments enhanced N uptake in various plants. For instance, upland rice treated with 8 mM NaCl showed increased N content [41], and *Arabidopsis* roots exposed to 5 and 10 mM NaCl exhibited elevated N levels [2]. The simultaneous increase in K^+^, N, Na^+^, and Cl^−^ content may have contributed to the enhanced cotyledon growth in Chinese white radish seedlings, possibly due to improved carbon (C) accumulation under low-NaCl conditions.

At 4 days old, Chinese white radish typically displays two heart-shaped cotyledons (Figure 1). These cotyledons may be crucial for photosynthetic processes, providing carbon sources for seedling growth and development. They have functions similar to those of mature leaves and play a vital role in photosynthesis. As shown in Figure 6, activities related to the Hill reaction, a marker of H_2_O oxidation during photosynthesis, also occur in these cotyledons. According to findings in *Arabidopsis thaliana*, the initiation of the photosynthetic process occurs when the first cotyledon is exposed [42]. However, it is well documented that the high accumulation of Na^+^ and Cl^−^ can lead to reduced levels of aminolevulinic acid (ALA), a precursor for chlorophyll synthesis [43]. Additionally, these conditions can enhance chlorophyllase activity, decreasing chlorophyll content and photosynthetic activity [44]. Our findings revealed the maintenance of Hill reaction ability in cotyledons under low NaCl concentrations, especially 10 mM (Figure 6A). Despite the large accumulation of Na^+^ and Cl^−^ in the leaves, theses ions might be compartmentalised in vacuoles [45]. Moreover, the assessment of scavenging activities through DPPH assays revealed a slight increase in cotyledons treated with 10 and 20 mM NaCl (Figure 6B), suggesting that plant cells can regulate free radical accumulation. This suggests that the use of 10 mM NaCl is more suitable than 20 mM NaCl for inducing Na^+^ and Cl^−^ accumulation in cotyledons, thereby maintaining the photosynthetic ability involved in oxygen evolution. These results are supported by the elevated expression levels of AQP–water gate proteins located on the cell membrane, specifically the *PIP1-1*, *PIP1-2*, *PIP2-1*, and *PIP2-2* genes (Figure 4), which facilitate the utilisation of free water as a substrate for this process.

## 4. Materials and Methods

Based on our preliminary experiment, the application of low concentrations of NaCl (10 and 20 mM) for 0–5 days promoted an increase in the fresh weight of the whole plant, including both the shoots and roots, of Chinese white radish (*Raphanus sativus* L. var. *longipinnatus* Bailey). In contrast, higher concentrations of 50 and 100 mM NaCl reduced these parameters by approximately 50% compared to the control (Figure 2). Moreover, the highest growth rate was observed in 4-day-old seedlings (see Supplementary Figure S1). The application of NaCl in the range of 0–100 mM also led to an increase in soil electrical conductivity (EC). EC values were measured using the 1:5 (soil/water) method and conductivity meter (Eutech, Vernon Hills, IL, USA), revealing a range of 0.08–0.15 dS/m in soils treated with 10 and 20 mM NaCl, which corresponds to slight salinity. In contrast, 50 mM NaCl resulted in EC values of 0.21–0.38 dS/m, indicating moderate salinity, while 100 mM NaCl led to EC values of 0.40–0.72 dS/m, also classified as high-salinity [18,46,47] (see Supplementary Figure S2). Therefore, NaCl concentrations of 10 and 20 mM were designated as low, while 50 and 100 mM were classified as moderate and high concentrations, respectively. Additionally, 4-day-old seedlings were selected as the optimal developmental stage for this study.

### 4.1. Plant Material and NaCl Treatments

The seedlings of 4-day-old Chinese white radish were prepared. The seeds were soaked in distilled water for 18 h, and 10 fully swollen seeds were selected for germination in a plastic container containing 200 g of fine sand. The sand cultures were initially watered with 40 mL of 5 NaCl solutions: 0 (control), 10, 20, 50, or 100 mM. Subsequently, 10 mL of the same concentration was applied daily to each treatment group. The cultivation of the seedlings was conducted in a greenhouse under a 12/12 h light/dark photoperiod at 30 ± 5 °C, a relative humidity of 70 ± 5%, and a photosynthetic photon flux density of 3000 ± 100 lux. Four-day-old seedlings were chosen for the analysis of growth, water status, AQP gene expression, ion contents, and physiology-related parameters.

### 4.2. Measurement of Growth Parameters

The percentage of germination was calculated using the following formula: germination (%) = (number of germinated seeds/number of sown seeds) × 100. Shoot heights and root lengths were measured using a ruler and recorded in centimetre intervals. The fresh and dry weights, which were obtained after drying the fresh plant samples at 80 °C for 2 days in hot air oven (Binder, Camarillo, CA, USA), of the cotyledons, hypocotyls, and roots, were determined using a precision balance (Ohaus, Parsippany, NJ, USA) and recorded in g. Cotyledon areas were determined by capturing images and utilising ImageJ (version 1.54g), a Java-based image processing programme. The measurements were recorded in mm^2^. Cotyledon thickness and palisade and spongy mesophyll cell sizes were determined under a light microscope (Olympus, Center Valley, PA, USA) using a cross-sectional technique and recorded in µm. Palisade mesophyll cells were measured in terms of width and length, whereas spongy mesophyll cells were measured only in terms of length or diameter.

### 4.3. Measurement of Water Status Parameters

The plant samples were first measured for fresh weight (FW). Subsequently, the samples were soaked in distilled water until they reached saturation or exhibited no change in fresh weight (approximately 4 h for the seedlings) to obtain their turgid weight (TW). Then, they were dried at 80 °C for 2 days to obtain the dry weight (DW). Following a method modified from Smart and Bingham [48], the relative water content (RWC) was calculated using the following formula: RWC (%) = [(FW − DW)/(TW − DW)] × 100. Following the modified method of Zeng et al. [49], the water content (WC) was calculated using the following formula: WC (g·g_DW_^−1^) = (FW − DW)/DW. And the degree of succulence (DS) was calculated using the following formula: DS (times) = FW/DW.

### 4.4. Measurement of Aquaporin Gene Expression

The expression of AQP genes (*PIP1-1*, *PIP1-2*, *PIP2-1*, *PIP2-2*, and *TIP1-1*) was determined using real-time polymerase chain reaction (real-time PCR). The fresh plant samples were ground in liquid nitrogen, and the frozen powder was mixed with TRIzol reagent (Invitrogen, Waltham, MA, USA) for 5 min to extract the mRNA. Chloroform was added, and the mixture was shaken and left to separate into two layers. The mixture was then centrifuged at 12,000 rpm for 15 min at 4 °C using a microcentrifuge (Hercuvan Laboratory Systems, Subang Jaya, Malaysia). The supernatant was transferred to a new tube, isopropanol was added, and the mixture was inverted and incubated at room temperature (25−30 °C) for 10 min. After centrifuging again at 12,000 rpm for 10 min at 4 °C, the supernatant was discarded. The mRNA pellet was washed with 70% ethanol, centrifuged at 10,000 rpm for 5 min at 4 °C, and the supernatant was discarded. The pellet was air-dried, incubated with molecular-grade water at −20 °C overnight, and then converted to cDNA using the Tetro cDNA Synthesis Kit (Bioline, Essex, UK) and PCR technique at 45 °C in a thermal cycler (Thermo Fisher Scientific, Waltham, MA, USA). The primers for the AQPs and GAPDH used in this study (Supplementary Table S1) were designed based on previously published sequences by Suga et al. [32,50], Tsuchihira et al. [51], Higuchi et al. [52], and Xu et al. [53]. GAPDH was selected as the reference gene for normalisation, as it has been identified as the most stable internal control for gene expression analysis across various radish organs under multiple conditions, including salt stress [54]. The cDNA concentration was measured at 260 nm using a nanodrop spectrophotometer (Thermo Fisher Scientific, Waltham, MA, USA). The expression of AQPs was detected using a Luna qPCR dye (New England Biolabs, Ipswich, MA, USA) and a real-time PCR system (QIAGEN, Hilden, Germany).at 59 °C.

The relative expression levels of the target genes were calculated using the 2^−ΔΔCt^ method [55]. The calculation was performed in the following steps:

1. ΔCt (Delta Ct) was obtained by subtracting the Ct (Cycle threshold) value of the reference gene from the Ct value of the target gene in each sample: ΔCt = Ct_AQPs_ − Ct_GADPH_
2. ΔΔCt (Delta Delta Ct) was calculated by subtracting the ΔCt of the control group from the ΔCt of the treatment group for each target gene: ΔΔCt = ΔCt_treatment_ − ΔCt_control_
3. The relative expression level of each gene was determined by the following equation: relative expression = 2^−ΔΔCt^

### 4.5. Measurement of Ion Contents

The cation contents, Na^+^, K^+^, Ca^+^, and Mg^+^, were determined using a method modified from that described by Hoo et al. [56]. The dried plant samples were digested in 1 N HNO_3_ until evaporation was complete. Subsequently, deionised water was added, and the resulting solution was filtered. The cation contents were quantified utilising a flame photometer (Cole-Parmer, Québec, QC, Canada), with the outcomes expressed in µmol·g_DW_^−1^.

The quantification of P (total phosphate; PO_4_^3−^), total N, extract S, C, and Cl^−^ was conducted using a technique modified from that outlined by the Science Center of Land Development [57]. For the analysis of total PO_4_^3−^, the dried plant samples were digested and completely evaporated in a 2:1 (*v*/*v*) mixture of HNO_3_ and HClO_4_. Subsequently, the sample was appropriately diluted with deionised water and Barton’s reagent (10.7 mM NH_4_VO_3_ and 21.5 mM (NH_4_)_6_Mo_7_O_24_·4H_2_O) was added during the digestion process to generate the yellow complex samples. The absorbance was measured at 420 nm using a spectrophotometer (BMG LABTECH, Ortenberg, Germany). The total PO_4_^3−^ was determined using a standard curve and recorded as a percentage of dry weight (%DW).

For the total N analysis, the dried plant samples were digested in H_2_SO_4_ (conc.) and the samples were appropriately diluted with deionised water. A Kjeldahl digestion apparatus with a mixed catalyst was used until the resultant samples became colourless solutions. Subsequently, 40% NaOH was introduced into the resulting samples, leading to the generation of ammonia (NH_3_). A mixing indicator and 3% boric acid (H_3_BO_3_) were used to facilitate the conversion of NH_3_ into ammonium dihydrogen borate (NH_4_H_2_BO_3_). The total N was determined in a titration process using H_2_SO_4_, used to convert NH_4_H_2_BO_3_ to ammonium hydrogen sulfate (NH_4_HSO_4_), and recorded as %DW.

Extract S analysis was conducted using the turbidimetric method. The dried samples were digested and evaporated using a 2:1 (*v*/*v*) mixture of HNO_3_ and HClO_4_. Subsequently, the sample was appropriately diluted with deionised water, and 2 M ammonium acetate (CH_3_COONH_4_) and 1g barium chloride (BaCl_2_·2H_2_O) were added to induce turbidity. The resulting turbidity was quantified by measuring the absorbance at 420 nm using a spectrophotometer. Extract S was subsequently calculated using a standard curve and expressed in nanomoles per gram of dry weight (nmol·g_DW_^−1^).

Carbon content was calculated using the organic matter by immersing a dried sample in a mixture of 1 N potassium dichromate (K_2_Cr_2_O_7_) and H_2_SO_4_ (conc.), followed by shaking overnight to convert the organic carbon into ammonium iron (*II*) sulfate hexahydrate (Fe(NH_4_)_2_(SO_4_)_2_·6H_2_O). An indicator (0.025 M O-phenanthroline ferrous sulfate indicator) was added, and 0.5 N ferrous ammonium sulfate was used to titrate the solution, resulting in a brown-red solution. The organic matter was calculated using the formula outlined by Walkley and Black [58] and recorded as %DW.

For Cl^−^ content analysis, the dried plant samples were mixed with calcium oxide (0.4 g CaO:1 g sample) and distilled water in a porcelain crucible and burned at 550 °C for 90 min. The samples were then allowed to cool overnight. The dry ash was washed multiple times with hot water and filtered. The Cl^−^ content was then measured using ion chromatography (Thermo Fisher Scientific, Waltham, MA, USA), and the results were calculated in µmol·g_DW_^−1^.

### 4.6. Measurement of Physiology-Related Parameters

The Hill reaction was performed using a method modified from Dean and Miskiewicz [59] and Latkowska et al. [60]. The fresh plant samples were ground in a grinding medium (0.04 M K_3_PO_4_, 0.008 M KCl, and 0.04 M sucrose at pH 6.8), filtered, and centrifuged at 4000 rpm for 5 min at 0 °C. The pellet was resuspended in the grinding medium. The chloroplast suspension was mixed with a 0.04 M phosphate buffer (pH 6.8), 0.1% 2,6-dichlorophenol indophenol (DCPIP), and distilled water. The absorbance was measured at 600 nm using a spectrophotometer as A_600S_ (using the chloroplast suspension without DCPIP as a blank). Subsequently, the chloroplast suspension was irritated with DCPIP under a 100 W light bulb at a 15 cm distance for 10 min, and the absorbance was measured again at 600 nm as A_600E_. The results were expressed as changes in OD. The activity of the Hill reaction was calculated using the formula [(A_600S_ − A_600E_)/A_600S_] × 100), and the outcomes were expressed as percentages of absorbance change (%absorbance change).

Free radical scavenging activity by the 2,2-diphenyl-1-picrylhydrazyl (DPPH) assay was determined using a method modified from Kumari et al. [61]. Plant extracts were prepared by grinding the fresh plant samples in 80% methanol (with 80% methanol used only as a blank) and filtering them through a filter paper. The plant extract was mixed with a 0.3 mM DPPH solution and incubated at room temperature for 10 min. The absorbance was measured at 517 nm using a spectrophotometer, and the results were calculated using the formula [(A_Blank_ − A_Sample_)/A_Blank_] × 100, as a percentage of inhibition (%inhibition).

The proline content was determined following the methods described by Bates et al. [62] and Ghoulam et al. [63]. The fresh plant samples were ground in 40% methanol, filtered, and treated with an acid ninhydrin reagent (0.25 g of ninhydrin dissolved in a 3:2 (*v*/*v*) mixture of glacial acetic acid and 85% orthophosphoric acid). The mixture was incubated in a water bath at 100 °C. The reaction was stopped by incubating the samples on ice, and 5 mL of toluene was added. The absorbance was measured at 528 nm using a spectrophotometer. The proline content was calculated using a standard curve and recorded in µmol·g_DW_^−1^.

### 4.7. Statistical Analysis

The experiments were conducted using a completely randomised design (CRD). Data from each experiment were statistically analysed using RStudio (version 2024.04.0+735). Analysis of variance (ANOVA), followed by Duncan’s multiple range test (*p* < 0.05), was performed using the ‘agricolae’ package (version 1.3-5). Pearson’s correlation coefficients were calculated using the base ‘cor()’ function, and correlation matrices were visualised using the ‘corrplot’ package (version 0.92).

## 5. Conclusions

Low concentrations of NaCl (10 and 20 mM) have been shown to enhance the growth of 4-day-old Chinese white radish seedlings, particularly in the cotyledon region. Among these concentrations, 10 mM NaCl proved more effective at promoting cotyledon development, leading to the increased accumulation of Na^+^, Cl^−^, K^+^, total nitrogen, carbon, and proline. This treatment also upregulated the expression of aquaporin (AQP) genes, notably *PIP1-1*, *PIP1-2*, *PIP2-1*, *PIP2-2*, and *TIP1-1*. Furthermore, low NaCl concentrations facilitated cell expansion, maintained Hill reaction activity, and enhanced free radical scavenging capacity. These physiological and biochemical changes collectively resulted in greater cotyledon area and thickness, as well as increased fresh and dry biomass.

These findings suggest that the application of 10 mM NaCl may serve as a simple, low-cost agronomic strategy to improve early seedling vigour and sprout biomass production in Chinese white radish. This approach could be particularly advantageous in controlled cultivation systems, such as hydroponics or greenhouses, where early growth is crucial for market value. Further research is recommended to elucidate the signalling mechanisms involved in AQP gene regulation under low-NaCl conditions. Moreover, understanding these responses could inform strategies to enhance seedling tolerance to abiotic stresses, such as water-deficit conditions.

## Data Availability

Data will be made available on request.

## Supplementary Materials

The following supporting information can be downloaded at: https://www.mdpi.com/article/10.3390/plants14111616/s1, Supplementary Figure S1: The effects of NaCl on the growth rate of fresh weight were evaluated after cultivating Chinese white radish seedlings for 5 days., Supplementary Figure S2: The electrical conductivity (EC) of the sand culture was measured using the 1:5 (soil/water) method after cultivating Chinese white radish seedlings for 5 days. Supplementary Table S1: Primers of AQP genes [32,50,51,52] and *GADPH* [53] used in this study.
