# Supplementary material for: Low NaCl Concentrations Increase Cotyledon Growth in Chinese White Radish (Raphanus sativus L. var. longipinnatus Bailey) Seedlings via Aquaporin-Mediated Water Transport
Source: Plants (Basel). 2025 May 26;14(11):1616. doi: 10.3390/plants14111616 (PMC12157832; doi:10.3390/plants14111616)
Supplement: Supplementary file 1 [file plants-14-01616-s001.zip › plants-3655800-supplementary.pdf]

Supplementary Materials

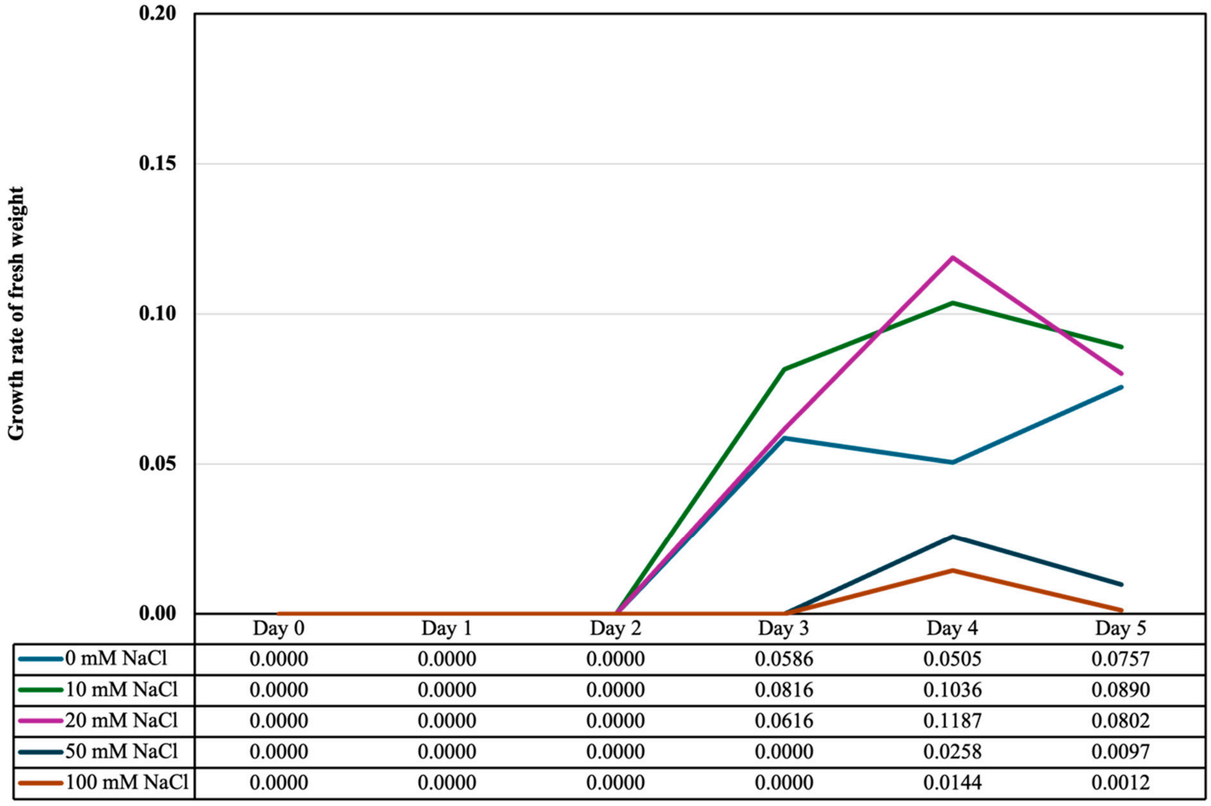

	Day 0	Day 1	Day 2	Day 3	Day 4	Day 5
0 mM NaCl	0.0000	0.0000	0.0000	0.0586	0.0505	0.0757
10 mM NaCl	0.0000	0.0000	0.0000	0.0816	0.1036	0.0890
20 mM NaCl	0.0000	0.0000	0.0000	0.0616	0.1187	0.0802
50 mM NaCl	0.0000	0.0000	0.0000	0.0000	0.0258	0.0097
100 mM NaCl	0.0000	0.0000	0.0000	0.0000	0.0144	0.0012

Supplementary Figure S1 The effects of NaCl on the growth rate of fresh weight were evaluated after cultivating Chinese white radish seedlings for 5 days. The graph above displays the data for plant fresh weight after the seedlings emerged from the sand culture

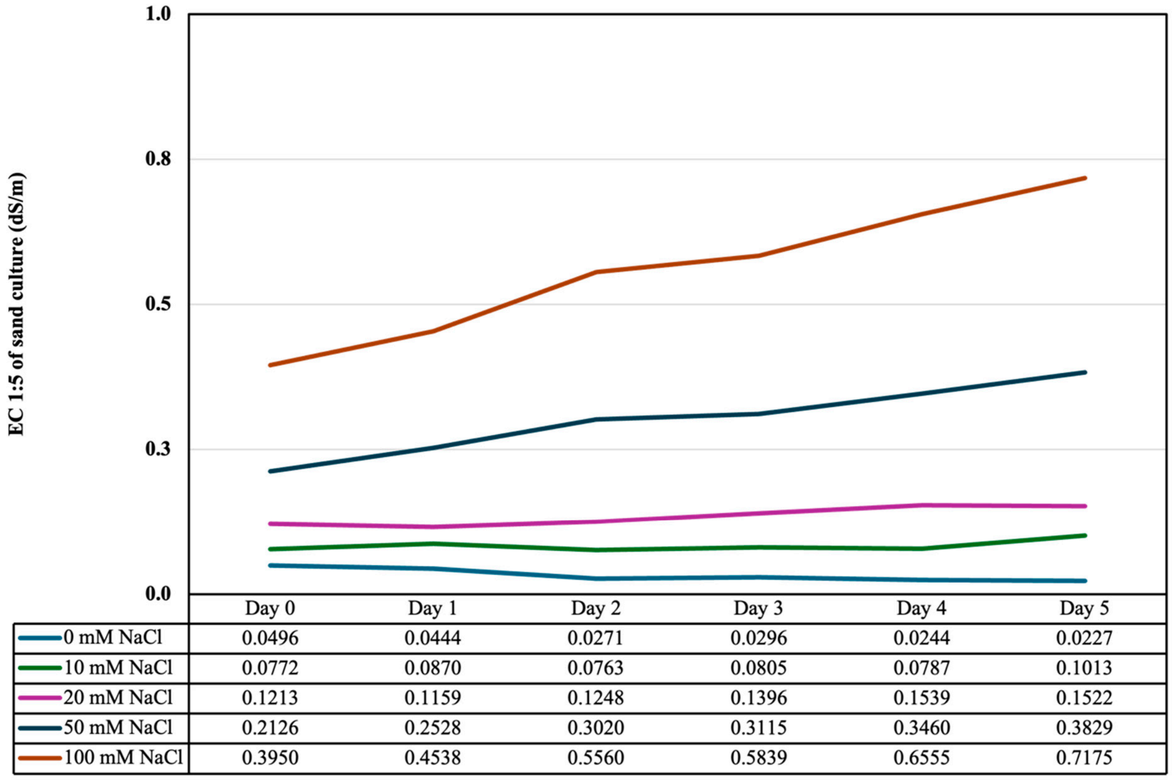

	Day 0	Day 1	Day 2	Day 3	Day 4	Day 5
0 mM NaCl	0.0496	0.0444	0.0271	0.0296	0.0244	0.0227
10 mM NaCl	0.0772	0.0870	0.0763	0.0805	0.0787	0.1013
20 mM NaCl	0.1213	0.1159	0.1248	0.1396	0.1539	0.1522
50 mM NaCl	0.2126	0.2528	0.3020	0.3115	0.3460	0.3829
100 mM NaCl	0.3950	0.4538	0.5560	0.5839	0.6555	0.7175

Supplementary Figure S2 The electrical conductivity (EC) of the sand culture was measured using the 1:5 (soil:water) method after cultivating Chinese white radish seedlings for 5 days.

Supplementary Table S1 The Primers of AQPs and *GADPH* gene which used in this study.

Primer	Primer Sequence ('5 to 3')	References
PIP1-1 (FORWARD)	AACCACCACCAGCTCCGTTTTT	[50]
PIP1-1 (REVERSE)	CAAGTGAACCAAGAAAACCGCA	[50]
PIP1-2 (FORWARD)	CAGGGGAATGTCTCTGACTCTG	[32]
PIP1-2 (REVERSE)	GGACCATTCATCGGTGCTGC	[32]
PIP2-1 (FORWARD)	TCTCGGATCTTTCAGAAGTGC	[51]
PIP2-1 (REVERSE)	AACACAAACAAGAAGATAACAAACTGG	[51]
PIP2-2 (FORWARD)	CCCCGAGTACACAAACATTCAC	[32]
PIP2-2 (REVERSE)	GGAGCTGCGATAGCTGCATTT	[32]
TIP1-1 (FORWARD)	GCATGCCGATCAGAAACATC	[52]
TIP1-1 (REVERSE)	ACGGTGTAAACAAGCCCGAATGT	[52]
GADPH (FORWARD)	GAAATCAAGAAGGCTATCAAGGAG	[53]
GADPH (REVERSE)	TTGTCACCAACGAAGTCAGT	[53]
